# Preoperatively-determined Red Distribution Width (RDW) predicts prolonged length of stay after single-level spinal fusion in elderly patients

**DOI:** 10.1016/j.bas.2024.102827

**Published:** 2024-05-04

**Authors:** Anton Früh, Dietmar Frey, Adam Hilbert, Claudius Jelgersma, Christian Uhl, Nitzan Nissimov, Peter Truckenmüller, David Wasilewski, Dimitrios Rallios, Matthias Hoppe, Simon Bayerl, Nils Hecht, Peter Vajkoczy, Lars Wessels

**Affiliations:** a Department of Neurosurgery, Charité – Universitätsmedizin Berlin, Corporate Member of Freie Universität Berlin, and Humboldt-Universität zu Berlin, and Berlin Institute of Health, Berlin, Germany; b CLAIM – Charité Lab for AI in Medicine, Charité Universitätsmedizin Berlin, Corporate Member of Freie Universität Berlin, Humboldt-Universität zu Berlin and Berlin Institute of Health, Charitéplatz 1, 10117, Berlin, Germany; c Medical Faculty Leipzig, Leipzig University, Leipzig, Germany; d BIH Biomedical Innovation Academy, BIH Charité Junior Digital Clinician Scientist Program, Charitéplatz 1, 10117, Berlin, Germany

**Keywords:** Frailty, Geriatrics, RDW, Spine surgery

## Abstract

**Introduction:**

Elderly patients receiving lumbar fusion surgeries present with a higher risk profile, which necessitates a robust predictor of postoperative outcomes. The Red Distribution Width (RDW) is a preoperative routinely determined parameter that reflects the degree of heterogeneity of red blood cells. Thereby, RDW is associated with frailty in hospital-admitted patients.

**Research question:**

This study aims to elucidate the potential of RDW as a frailty biomarker predictive of prolonged hospital stays following elective mono-segmental fusion surgery in elderly patients.

**Material and methods:**

In this retrospective study, we included all patients with age over 75 years that were treated via lumbar single-level spinal fusion from 2015 to 2022 at our tertiary medical center. Prolonged length of stay (pLOS) was defined as a length ≥ the 3rd quartile of LOS of all included patients. Classical correlation analysis, Receiver-operating characteristic (ROC) and new machine learning algorithms) were used.

**Results:**

A total of 208 patients were included in the present study. The median age was 77 (IQR 75–80) years. The median LOS of the patients was 6 (IQR 5–8) days. The data shows a significant positive correlation between RDW and LOS. RDW is significantly enhanced in the pLOS group. New machine learning approaches with the imputation of multiple variables can enhance the performance to an AUC of 71%.

**Discussion and conclusion:**

RDW may serve as a predictor for a pLOS in elderly. These results are compelling because the determination of this frailty biomarker is routinely performed at hospital admission. An improved prognostication of LOS could enable healthcare systems to distribute constrained hospital resources efficiently, fostering evidence-based decision-making processes.

## Highlights

• RDW serves as a predictor of prolonged hospital stay in elderly patients following single-level fusion.
• Novel machine learning approaches improve prediction of prolonged length of stay.
• Lumbar spinal fusion can be performed safely in elderly patients.

## 1 Introduction

Lumbar spine surgeries are frequently performed interventions (Bundesamt, 2020). Thereby the total costs of surgical interventions annularly amounted to about 1.6 billion USD hospital expenses in the United States (Deyo et al., 2010). Increasing life expectancy has resulted in a globally rising number of elderly patients undergoing spinal surgeries (Fehlings et al., 2015). Besides minimally invasive procedures, especially number of fusion surgeries in elderly patients is further growing. In this cohort, the incidence of preexisting and secondary diseases is high, leading to an overall acceptable but elevated risk profile regarding complications (Fehlings et al., 2015; Klimov et al., 2022; Imajo et al., 2017). In this evolving topic, the concept of frailty has emerged as a potent prognostic outcome determinant. Frailty is a multifactorial syndrome characterized by decreased physiological reserve and increased vulnerability to stressors, potentially leading to adverse clinical outcomes and more complications (Makary et al., 2010). Beyond the assessment of various indices (Pritchard et al., 2017; Hamaker et al., 2012), which lead to additional diagnostic efforts, attempts are made to more simply assess the frailty of patients using indirect methods, such as the measurement of diverse muscle thicknesses (Liao et al., 2019) or blood biomarkers (Picca et al., 2022). Thereby, spinal fusion surgeries can be particularly challenging for frail patients, as they often involve high physiological stress demands, longer operation times, and increased risk of postoperative complications (Panayi et al., 2019). Thus, there is an urgent need better to understand the role of frailty in spinal surgery and to develop strategies for optimizing patient care.

The red blood distribution width (RDW) is a routinely determined laboratory parameter that quantifies the variability in erythrocyte volumes (Salvagno et al., 2015a). It has been identified as a potential predictive marker that may serve as independent risk factor for the severity and progression of various conditions, including cerebrovascular diseases (Li et al., 2017), proinflammatory diseases, and traumatic brain injury (Li et al., 2017; Lin et al., 2023; Lorente et al., 2021). This suggests that RDW might indirectly serve as a routine biomarker for frailty. Given this context, we hypothesized that RDW could be indicative of increased frailty in elderly patients undergoing elective mono-segmental fusion surgery. Our objective was to examine the predictive capability of RDW in determining the length of stay (LOS) in the hospital for elderly patients receiving single-level spinal fusions due to a degenerative disease.

## 2 Materials and methods

This is a retrospective single-center study. Ethical approval was granted by the local ethics committee of Charité University Hospital (EA4/159/23). We included all patients with age over 75 years that were treated via lumbar single-level spinal fusion from 2015 to 2022. Spondylodiscitis, traumas, rheumatic disorders, current malignant neoplasms, history of psychiatric illness, alcohol or drug dependency, and psychotropic medication were defined as exclusion criteria. Only elective cases were included. All patients received a transforaminal lumbar interbody fusion. Medication for anticoagulation and/or platelet inhibition was discontinued 1 week prior to surgery. Standard blood samples were drawn from all patients at hospital admission (maximum 72 h prior to the intervention). Length of stay (LOS) was defined as the day from surgery till the day of discharge of the patient. According to previous studies (Saravi et al., 2022) prolonged LOS was defined as a length ≥ the 3rd quartile of LOS of all included patients.

Statistical analysis was performed with SPSS version 25 (IBM Corp), Microsoft Excel 2021, and GraphPad Prism 8.4.2. Discrete data were presented as counts and percentages and analyzed by using the chi-square test. Continuous data were presented as the median and interquartile range (IQR) and compared using Mann-Whitney statistics. Two-sided p-values <0.05 were taken to indicate statistical significance. Machine learning calculations were performed on a Python-based already published and used framework (Zihni et al., 2020; Dengler et al., 2021). The Python code can be accessed on GitHub (https://github. com/prediction2020/explainable-predictive-models). This study uses calculations based on a plain GLM, an L1 regularized GLM, and a GLM elastic net with an additional L2 regularization (ElasticNET). In addition, tree boosting algorithms, multilayer perceptron (MLP) artificial neural nets and Naive Bayes (NB) classifiers were performed. Feature importance ratings were determined using Shapley Additive exPlanations (SHAP) values. To minimize potential confounding effects, variance inflation factor (VIF) was applied to assess multicollinearities for all features. Data were split randomly into training and test sets with a ratio of 4 to 1. The mean/mode imputation and feature scaling using zero-mean unit variance normalization based on the training set was performed on training and test sets. Hyperparameter tuning was performed with 10-fold cross-validation. The whole process was repeated in 50 shuffles. Model performance was determined via receiver operating characteristic (ROC)-analysis by measuring the area under the curve (AUC). All measures are presented as medians over all shuffles. Absolute values of the SHAP feature importance scores were scaled to the unit norm and, for each of the 50 shuffles, rescaled to a range from zero to one with their sum equal to one. Each mean and standard deviation, calculated on the test sets over all shuffles, were included in the final rating measurement.

## 3 Results

### 3.1 Demographics

208 patients were included in the present study. The median age of the study population was 77 years (75–80), and 58.2 % of the patients were female. The median LOS of the patients was 6 (Imajo et al., 2017; Makary et al., 2010; Pritchard et al., 2017; Hamaker et al., 2012) days. Therefore, patients that stayed 8 days (=3rd quartile) or longer after surgery were categorized into the prolonged LOS (pLOS) group. Patients with shorter stays were allocated to the normal LOS (nLOS) group. The baseline characteristic of the study population is presented in Table 1.

**Table 1:** Baseline characteristics of the study population stratified between nLOS and pLOS group.

	Total study population (n = 208)	nLOS (n = 138)	pLOS (n = 70)	p-value
Age, years, median (IQR)	77 (75–80)	77 (75–80)	77 (73–80)	n.s. (0.751)
Female sex, n (%)	121 (58.2)	77 (55.8)	44 (62.9)	n.s. (0.329)
BMI, kg/m^2^. median (IQR)	27.3 (24.2–30.5)	27.4 (24.2–30.4)	26.5 (24.2–30.5)	n.s. (0.747)
**Medical history**				
Diabetes, n (%)	23 (11.1)	16 (11.6)	7 (10.0)	n.s. (0.729)
Hypertension, n (%)	67 (32.2)	44 (31.9)	23 (32.9)	n.s. (0.887)
Coronary artery disease, n (%)	14 (6.7)	7 (5.1)	7 (10.0)	n.s. (0.180)
Anticoagulative medication, yes, n (%)	62 (29.8)	39 (28.3)	23 (32.9)	n.s. (0.493)
**Preoperative ASA score:**	–			**sig (0.011)**
1, n (%)	8 (4.3)	6 (4.8)	2 (3.2)	
2, n (%)	104 (55.6)	78 (62.4)	26 (41.9)	
3, n (%)	73 (39.0)	41 (32.8)	32 (51.6)	
4, n (%)	2 (1.1)	0 (0.0)	2 (3.2)	
**Laboratory values at admission (routine lab)**				
Sodium, mmol/l, median (IQR)	141 (138–144)	140 (138–142)	141 (138–143)	n.s. (0.161)
Potassium, mmol/l, median (IQR)	4.2 (3.9–4.4)	4.2 (4.0–4.4)	4.1 (3.8–4.4)	n.s. (0.094)
Clucose, mg/dl, median (IQR)	109 (96–135)	107 (95–135)	113 (97–143)	n.s. (0.449)
Creatinine, mg/dl, median (IQR)	0.91 (0.78–1.11)	0.93 (0.78–1.11)	0.87 (0.76–1.13)	n.s. (0.371)
CRP, mg/l, median (IQR)	2.2 (0.8–5)	1.7 (0.7–3.4)	4.0 (10.2)	**sig (0.001)**
TSH, mU/l, median (IQR)	1.4 (0.9–2.3).	1.2 (0.8–2.2)	1.63 (1.1–2.8)	**sig (0.027)**
Hb, g/l, median (IQR)	13.3 (12.4–14.3)	13.4 (12.7–14.4)	12.9 (11.1–14.0)	**sig (0.002)**
Hk, l/l, median (IQR)	0.39 (0.36-0-42)	0.39 (0.37–0.42)	0.38 (0.34–0.41)	**sig (0.003)**
Erythrocytes,/pl, median (IQR)	4.4 (4.1–4.7)	4.4 (4.2–4.7)	4.4 (3.7–4.7)	n.s. (0.084)
Leucocytes,/nl, median (IQR)	7.6 (6.5–9.1)	7.6 (6.3–9.1)	7.5 (6.6–8.7)	n.s. (0.875)
Thrombocytes,/nl, median (IQR)	249 (199–297)	251 (197–298)	249 (208–298)	n.s. (0.681)
MCV, fl, median (IQR)	89 (87–92)	90 (88–93)	88 (86–91)	**sig (0.003)**
MCH, pg, median (IQR)	30.4 (29.5–31.6)	30.8 (29.7–31.6)	29.8 (28.9–31.2)	**sig (0.003)**
MCHC, g/dl, median (IQR)	33.9 (33.3–34.6)	33.9 (33.4–34.6)	34 (33.0–34.7)	n.s. (0.276)
MPV, fl, median (IQR)	10.5 (9.8-11-1)	10.5 (9.8–11.1)	10.5 (10.0–11.1)	n.s. (0.823)
RDW-CV, %, median (IQR)	13.5 (12.9–14.1)	12.7 (13.4–13.8)	13.8 (13.2–14.6)	**sig (p < 0.001)**
Quick, %, median (IQR)	98 (90–105)	100 (92–107)	95 (85–102)	**sig (0.003)**
INR, median (IQR)	1.01 (0.97–10.07)	1 (0.96–1.1)	1.03 (0.99–1.1)	**sig (0.006)**
apTT, seconds, median (IQR)	32.2 (30.1–35.1)	31.6 (29.7–34.3)	33.9 (31.2–37.4)	**sig (0.001**)

Abbreviations: ASA = American Society of Anesthesiologists, n = number, IQR = interquartile range, pLOS = prolonged length os stay (8 days or longer), nLOS = normal length of stay (<8 days).

### 3.2 Outcome

**Fig. 1:**
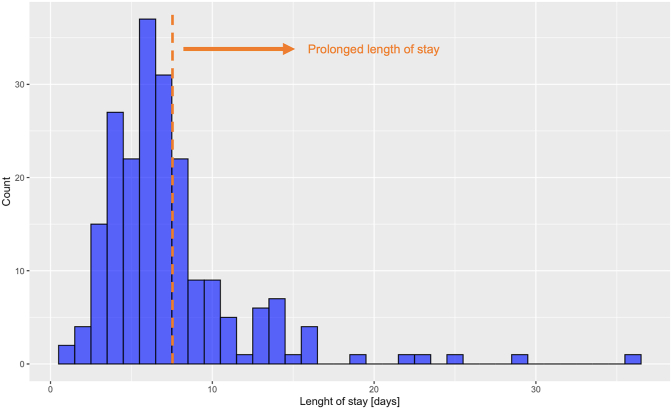
Distribution of length of stay.

The frequency distribution of the LOS in the study population is presented in Fig. 1. The data show a skewed distribution. Most patients stayed 6 days at the hospital. Overall, 145 (69.7%) patients could be discharged at home and 63 (30.3%) patients had to be transferred to a secondary hospital or rehab.

The median duration of the surgery was 171 (130–207) minutes. The perioperative mortality until the time-point of discharge was 0%. Intraoperative complications occurred in 5.3 % (n = 11) of the patients. In detail, 9 patients suffered from accidental dural tears that were treated directly via suture. One patient presented a transient intraoperative tachyarrhythmia, and 1 patient suffered from extensive intraoperative bleeding with the need for substitution of blood products. None of the patients suffered from persistent morbidity after the surgery.

### 3.3 Influence of laboratory parameters on length of stay

**Fig. 2:**
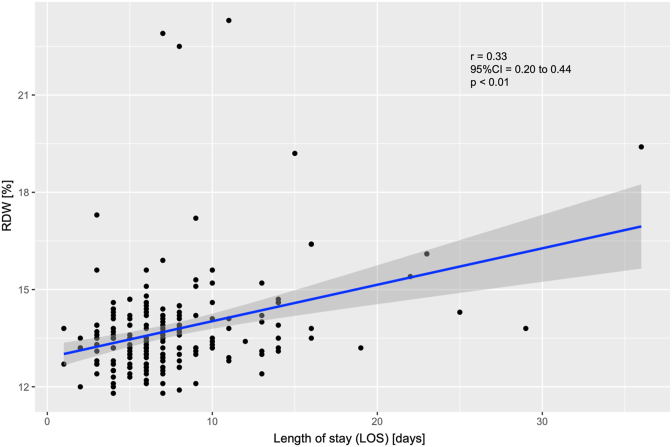
Correlation between RDW at admission and LOS (*p < 0.001). LOS = length of stay (days), RDW = Red blood distribution width (%), r: Correlation coefficient, CI: Confidence interval.

**Fig. 3:**
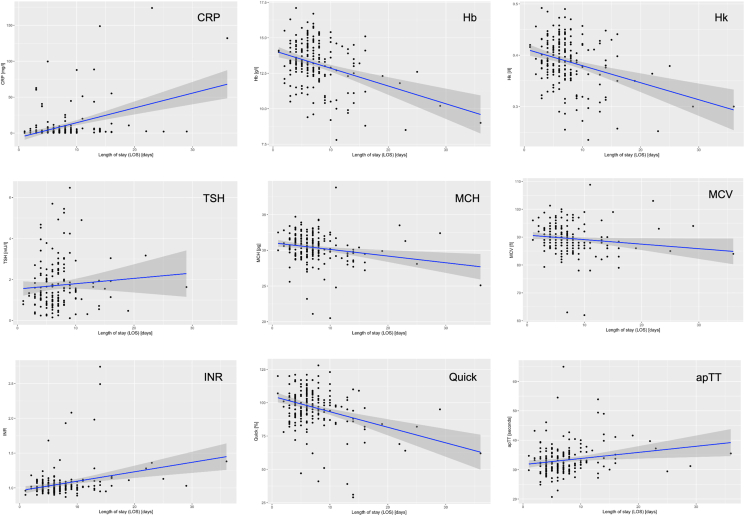
Correlation diagrams of admission laboratory values. The corresponding results of the statistical analysis are presented in Table 2.

There was a significant positive correlation between LOS and RDW, r = 0.33, p < 0.01, 95% CI [0.20, 0.44]. Fig. 2 presents the associated correlation diagram. pLOS and nLOS group show significant differences between RDW (*p < 0.01), CRP (*p = 0.01), MCH (*p = 0.0027), TSH (*p = 0.027), Hb (*p = 0.002), Hk (*p = 0.003), MCK (*p = 0.003), Quick (*p = 0.003), INR (*p = 0.003) and apTT (*p = 0.01) at admission. The correlation diagrams und statistical results between the significant admission laboratory values and LOS are provided in Fig. 3 and Table 2.

**Table 2:** Pearson product-moment correlation analysis between laboratory values and LOS.

variable	r	95%CI	p-value
correlation between LOS and			
RDW	0.33	0.20 to 0.44	**< 0.01**
CRP	0.40	0.27 to 0.51	**< 0.01**
Hb	−0.35	−0.46 to −0.22	**< 0.01**
Hk	−0.32	−0.43 to −0.19	**< 0.01**
TSH	0.08	−0.08 to 0.23	0.32
MCH	−0.20	−0.33 to −0.07	**< 0.01**
MCV	−0.14	−0.27 to −0.002	**0.046**
INR	0.28	0.15 to 0.41	**< 0.01**
Quick	−0.34	−0.46 to −0.21	**< 0.01**
apTT	0.18	0.04 to 0.32	**0.01**

Abbreviations: r: Correlation coefficient, CI: Confidence interval.

**Fig. 4:**
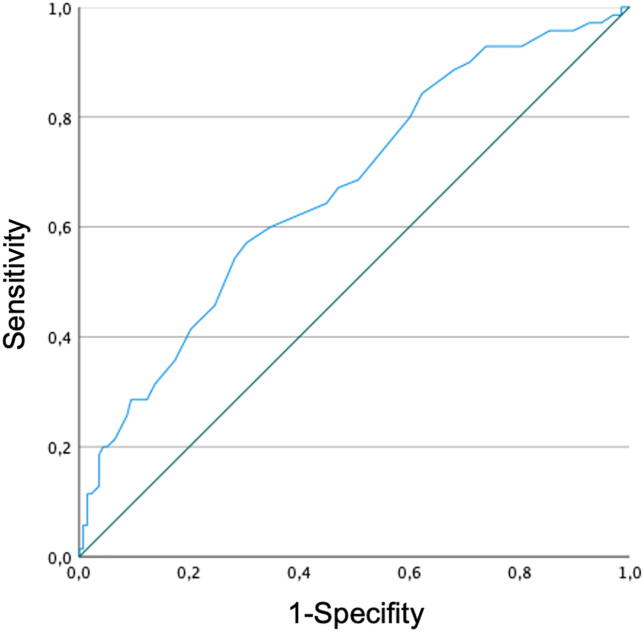
ROC-Curve for the prediction of pLOS with imputation of single RDW values.

To evaluate the prediction performance of RDW for prolonged stay a Receiver-operating characteristic (ROC) analysis with imputation of single RDW values was performed. This calculation shows an Area under the curve (AUC) of 67 %. The correlating ROC-Curve is presented in Fig. 4. Calculation of Youden-Indices reveal an optimum cut-off value for RDW at 13.7 %, with a corresponding specificity of 70% and a sensitivity of 57% for the prediction of pLOS.

Univariate analysis revealed RDW, CRP, TSH, Hb, Hk, Erythocytes, MCH, Quick, INR and apTT as risk factors for pLOS. The results are provided in Table 3.

**Table 3:** Univariate model for risk factors associated with prolonged length of stay.

variable	OR	95%CI	p-value
Sodium, mmol/l, median (IQR)	1.05	0.97 to 1.14	0.223
Potassium, mmol/l, median (IQR)	0.54	0.26 to 1.14	0.106
Clucose, mg/dl, median (IQR)	1.00	0.99 to 1.01	0.396
Creatinine, mg/dl, median (IQR)	0.85	0.37 to 2.00	0.718
CRP, mg/l, median (IQR)	1.03	1.01 to 1.04	**0.008**
TSH, mU/l, median (IQR)	1.37	1.04 to 1.80	**0.023**
Hk, l/l, median (IQR)	0.00	0.00 to 0.10	**0.001**
Erythrocytes,/pl, median (IQR)	0.51	0.29 to 0.88	**0.015**
Leucocytes,/nl, median (IQR)	1.02	0.91 to 1.15	0.692
Thrombocytes,/nl, median (IQR)	1.00	1.00 to 0.01	0.362
MCV, fl, median (IQR)	0.94	0.89 to 1.00	0.053
MCH, pg, median (IQR)	0.86	0.74 to 0.99	**0.036**
MCHC, g/dl, median (IQR)	0.78	0.59 to 1.04	0.093
MPV, fl, median (IQR)	0.97	0.83 to 1.12	0.650
RDW-CV, %, median (IQR)	1.52	1.17 to 1.97	**0.002**
Quick, %, median (IQR)	0.97	0.95 to 0.99	**0.001**
apTT, seconds, median (IQR)	1.08	1.02 to 0.15	**0.009**

Abbreviations: IQR: Interquartile range.

**Fig. 5:**
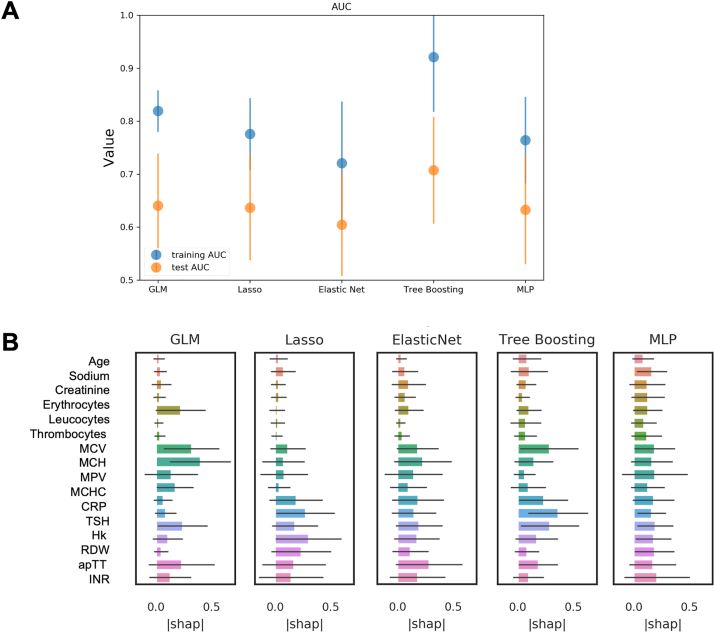
AI-based prediction of prolonged hospital stay. A. Graphical representation of the performance and feature rating for the combined feature set. Tree boosting (Catboost) models reached the highest AUC in training and test sets. B Feature rating of included parameters. Abbreviations: AUC – area under the curve, MLP – multilayer perceptron.

Further the prediction performance of prolonged LOS based in multiple variable inclusion with ne use of novel AI prediction models was evaluated. The results of these machine-learning approaches are presented in Fig. 5. The best AUC of the training samples was 0.92 (Tree Boosting algorithm). The best AUC of the test samples was 0.71 (Tree Boosting algorithm). The data reveals that, among others, RDW, CRP, TSH, and MCH play an important role in the multivariate AI based prediction of pLOS.

### 3.4 Influence on type of discharge

Patients that had to be transferred to a secondary hospital or rehab after the surgical treatment show significantly higher preoperative levels of RDW compared to patients that could be discharged at home (Median RDW_DischargeAtHome_ = 13.3 (IQR 12.7–13.8) %, RDW_DischargeAtHospital/Rehab_ = 13.8 (IQR 13.2–14.6), p* = 0.035). ROC Analysis shows a predictive performance for the need of a transfer to a secondary hospital or rehab with an AUC of 60%. Machine learning approaches bases on multivariable imputation show best performances with Tree Boosting algorithms. Thereby, AUC_TreeBoosting_ of the training samples was 0.91 and of the test samples 0.65.

## 4 Discussion

The novel finding of the study is that RDW may serve as a predictor for a prolonged length of hospital stay in elderly patients. These results are compelling because in contrast to other frailty markers, the preoperative determination of RDW does not imply additional financial or logistical requirements since it is routinely performed at hospital admission. A better prediction of LOS may help health systems to optimally allocate limited hospital resources and make informed, evidence-based decisions.

LOS is an established parameter evaluating socioeconomic costs, surgical success, complications, and outcome (Epstein et al., 2011). Various studies showed that additional days at hospitals after surgeries are associated with significantly increased expenditures for hospitals (Saravi et al., 2022; Weinstein et al., 2006). *Klineberg* et al. showed that long-term hospital patients suffering from spine deformity could cause additional costs of up to 19,000 USD compared to patients with a shorter hospital stay. They further showed that the age of the patients, numbers of fused levels, infections, and comorbidities are risk factors for a higher death rate after surgery but failed to predict prolonged stay at the hospital (Klineberg et al., 2016). *Saravi* et al. developed an AI-based algorithm to predict the length of stay after lumbar decompression surgery; thereby, risk factors for prolonged stay were the age of the patients, CRP, and the duration of surgery (Saravi et al., 2022).

Preoperative predicting of extended LOS allows hospitals to assess the overall patient load, which allows better scheduling of patient admissions, leading to a reduced variation of bed occupancies in hospitals. Moreover, it establishes the basis for the possibility of implementing specialized preoperative preparation protocols for these identified patients. Our results show for the first time that RDW exhibits moderate predictive performance (AUC = 67 %) in predicting prolonged hospital stays in elderly patients, that were treated with a single-level spinal fusion. Utilizing novel machine learning techniques and incorporating additional laboratory values led to a modest enhancement in multivariate prediction performance (AUC_Catboost, training_ 92 %; AUC_Catboost, test_ = 72%). Furthermore, the study shows significant increased levels of RDW in patients with the need of a transfer to a secondary hospital or rehab. However, the prediction performance is weak, both based on single RDW imputation (AUC = 60%) and based on multivariate machine-learning approaches (AUC_Catboost, test_ = 65%).

The data from the present study exhibiting a mortality rate of 0%, substantiate the premise that lumbar spinal fusions can be safely performed in an elderly patient collective. However, the frailty status can significantly influence surgical outcomes and complications. *Alkare* et al. have shown that high RDW values are associated with frailty in elderly patients at an emergency departments and are associated with a higher mortality-rate (Alakare et al., 2023). The authors think that it might be beneficial to include RDW in risk stratification models of elderly patients' prior lumbar fusion surgery to identify patients with extended lengths of stay. This may also guide shared decision-making about whether to proceed with surgery or consider non-surgical management options.

Several hypotheses exist for how erythrocyte heterogeneity is associated with frailty and adverse outcomes in patients (Alakare et al., 2023). These include compromised erythropoiesis and reduced lifespan of red blood cells due to organ dysfunction, metabolic disturbances, oxidative stress, and inflammatory responses, which lead to variations in red cell volumes. Furthermore inadequate nutrition and erythrocyte fragmentation have been identified as potential causes for elevated RDW. Also a long-term clinical deterioration due to telomere shortening, indicative of cellular ageing is correlating with the mean corpuscular volume may be reflected in elevated RDW levels (Alakare et al., 2023; Salvagno et al., 2015b; Pan et al., 2019; Kim et al., 2022).

This study is inherently limited due to its retrospective study design. The data demonstrated a significant, albeit modest (r = 0.33), correlation between length of stay (LOS) and red cell distribution width (RDW), along with an area under the curve (AUC) of 67% for predicting prolonged LOS (pLOS). Thus, while there is a statistical association between RDW and prolonged hospital stay, further studies are necessary to confirm the robustness of the data, ensuring that RDW can be considered a reliable predictor on its own. Moreover, the RDW parameter can be influenced by numerous conditions such as thyroid disorders, deficiencies of B12 and iron, bone marrow dysfunction, and inflammatory diseases. Consequently, an inherent bias originating from these alterations cannot be categorically excluded. Further complexities arise due to potential influences from medication usage and renal and hepatic dysfunctions. Finally, it cannot be ruled out that available capacities in the subsequent clinics may have influenced the LOS at the hospital.

## 5 Conclusion

RDW may serve as a predictor for a prolonged length of hospital stay in elderly patients that are treated via one-level lumbar fusion surgery. These results are particularly compelling because the determination of this frailty biomarker is routinely performed at hospital admission. An improved prognostication of Length of Stay (LOS) could enable healthcare systems to efficiently distribute constrained hospital resources, fostering evidence-based decision-making processes.

## Statement and declarations

The authors have nothing to declare and there is no potential conflict.

## Declaration of competing interest

We confirm that the manuscript complies with all instructions to authors, further we confirm that the authorship requirements have been met and the final manuscript was approved by all authors. We confirm that this manuscript has not been published elsewhere and is not under consideration by another journal. Lead ethics approval was approved and the study was performed according to the Declaration of Helsinki. There is no conflict of interest of any of the authors. The study received no funding. Dr. Früh funded by the BIH Charité Junior Digital Clinician Scientist Program of the Charité – Universitätsmedizin Berlin, and the Berlin Institute of Health at Charité (BIH).
